# Mental Exercises Necessary to Health

**Published:** 1885-03

**Authors:** 


### MENTAL EXERCISES NECESSARY TO HEALTH.

"The improvement of the memory is a familiar instance of an increase of mental power produced by exercise; and the beating sense of fulness and quickened circulation in the head induced by intense study or thought, shows that an organic process goes on when the brain is in activity, similar to that which takes place in the muscular system under exercise. On the contray, when the organ is little used, little expenditure of its power and substance takes place, little blood and little nervous energy are expended for its support, and therefore little is sent; nutrition in consequence soon becomes languid and strength impaired. To all these laws, the brain is subject equally as the rest uf the body. Frequent and regular exercise gives it increased susceptibility of action, with power to sustain it, the nervous energy requiring strength as well as the vascular. Disease of its functions, or, in other words, inactivity of intellect and of feeling impairs the structure and weakens the several powers which it serves to manifest. The brain, therefore, in order to maintain its healthy state, requires to be duly exercised."—*Barlow on Physical Education.*

